# Knowledge, attitude and practices about dengue fever among adults living in Pwani Region, Tanzania in 2019

**DOI:** 10.4314/ahs.v20i4.12

**Published:** 2020-12

**Authors:** Method Kazaura

**Affiliations:** Muhimbili University of Health and Allied Sciences

**Keywords:** Attitude, Dengue, knowledge, practice, rural

## Abstract

**Background:**

Dengue fever (DF) is currently widespread in tropical and sub-tropical countries. Among the triggers of epidemic include urbanization and internal migrations. Within the past few years, there have been DF outbreaks in Tanzania. Although Pwani region is among the predicted risk areas for the DF, there is insufficient data about people's knowledge, attitude and practices towards prevention of DF in their settings. Therefore, the aim of this study was to assess knowledge, attitude and practices about DF among adults in Pwani region in Tanzania.

**Methods:**

The cross-sectional study conducted in Mkuranga District, Pwani region in Tanzania. We used face-to-face interviews to collect data. The main analytical procedure was descriptive using frequencies.

**Results:**

The majority, 97.7%, were aware of DF. Nevertheless, almost 80% had a low knowledge on symptoms, transmission and vector control measures. Furthermore, less than 20% had positive attitude towards dengue fever prevention, severity of the illness and health seeking behavior.

**Conclusion:**

Lack of enough knowledge and positive attitude about disease transmission, symptoms and preventive measures put the population at high risk of contracting the disease. There is need to create and improve friendly, correct and simple information, education and education messages for the rural populations.

## Introduction

Dengue fever (DF) is a mosquito-borne viral infection that is caused by a virus of the *Flaviviridae* family with four (DEN-1, DEN-2, DEN-3 and DEN-4) distinct serotypes. Transmission of the virus to humans occur through a bite of an infected female *Aedes aegypti* during the day time and night^1^,^2^. DF is characterized by severe illness with several symptoms including fever, at least 40°C/104°F, severe headache, nausea, vomiting, swollen glands, pain behind eyes, fatigue, severe abdominal pains and some other symptoms that mimic that of malaria symptoms^3^,^4^,^5^. The primary vector of DF is the *Aedes aegypti* mosquito which is more widely dispersed now than at any time in the past, pacing billions of humans at risk of infection with one or more of the four-dengue serotypes^6^. Patients with DF experience high medical costs and severe health deterioration, especially in psychological dimensions^7^. Although DF has some similarities with malaria in terms of causing acute illness, malaria may be chronic while DF cannot. Nevertheless, there are more advanced clinical features to distinguish DF from other infections^8^.

The outbreak of DF is currently widespread in tropical and sub-tropical countries, primarily because of climatic changes associated with global warming and heavy rains^9^,^10^. Although Pwani region is predominantly rural, *Aedes* mosquitoes are likely to find habitat due to the swampy environment, bushes, livestock keeping and a climatic conducive for the *Aedes aegypti*; making the region among the predicted risk areas for the DF. The conducive environments for breeding sites of *Aedes aegypti* mosquitoes include some areas in urban centers turned into agriculture, flowerpots, discarded tires and sinks; jerricans, plastic drums and other containers around the residence^11^,^12^. Because of the diverse breeding habitats, the populations of *Aedes* mosquito can increase during and after heavy rainfalls; this, coupled with the habit of taking multiple blood meals from multiple human hosts serves as a risk factor for the outbreaks of DF.

Within this decade, there have been several case notifications of DF in sub-Sahara African countries^13^. Although accurate data on the incidence or prevalence of DF in most of the sub-Saharan countries are either missing or inaccurate, more than 60% of the population in Africa is at risk of dengue and the disease has so far affected almost 40 countries in the region^14^,^15^. Over the years, there have been estimates of DF prevalence in some parts of Africa ranging between 8 and more than 70%^16^–^18^. Nevertheless, an in-depth meta-analysis of DF in sub-Saharan Africa estimates the prevalence of DF to be 15.6% and 24.8% among apparent healthy and febrile individuals respectively between 2000 and 2007^19^. In Tanzania, the prevalence of DF was between 0.5% and 50.6% in the past two decades^19^. In addition, there have been reports of outbreaks between 2015 and 2020 along the eastern coast of mainland Tanzania and in Zanzibar^4^,^20^–^24^. Although the prevalence of DF in Pwani region is not well known, there was an outbreak of DF in the Eastern belt of Tanzania that includes Pwani, Dar es Salaam and Morogoro regions in May and June 2019.

At present, unlike malaria, DF has no cure except to combat its early symptoms. In some countries, there is relatively high rate of acceptance and amount of willingness to pay for the dengue vaccine among patients with DF^25^. The preventive measures against *Aedes*-borne diseases like DF among individuals, communities and policy makers remain as the best course of action^26^. In rural areas, these measures include to prevent daytime mosquito bites in public places by fumigation as well as controlling breeding sites.

While it is very important to properly manage the symptoms, prompt and correct detection of the symptoms are among vital elements to reduce the disease severity and fatality^27^. In less developed nations, especially in rural settings, lack of basic education, correct understanding of DF symptoms different from other infections like malaria compounded by poor health seeking behavior mystify the patients and their care-takers. It becomes evident when there is lack of knowledge, not only among the community, but also to health care providers on other causes of acute febrile illnesses apart from malaria to warrant misconceptions and negative attitude towards DF^28^.

There is lack of data about the knowledge, attitude and practices towards DF among community members in Pwani Region. The present study aimed at assessing these parameters to come up with data that may be used to strategize preventive and control measures against DF.

## Materials and methods

### Study design and settings

**Figure 1:**
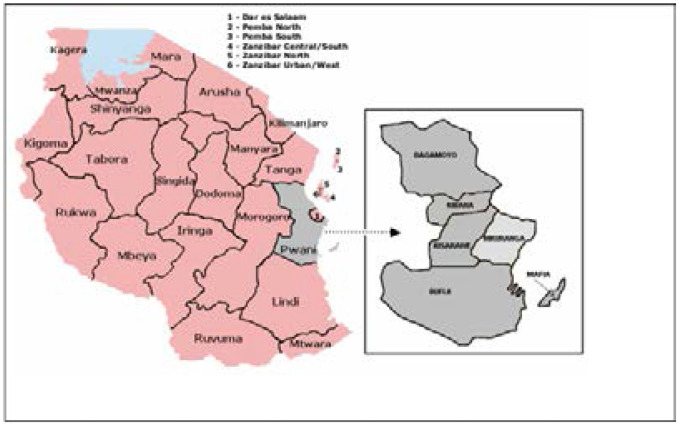
Map of Tanzania with Mkuranga (Source: Torell, et al. 2006^30^)

This was a cross-sectional descriptive study conducted in July 2019. Since we aimed to study knowledge, attitude and practices on DF specifically among rural dwellers, we conducted the study in the Pwani region. We purposefully selected Pwani because it is a predominantly rural region but at high risk of DF. The region has seven districts (Kibaha, Bagamoyo, Kisarawe, Mafia, Rufiji, Kibiti and Mkuranga). We randomly selected Mkuranga District. The District (Figure 1) is located on the Eastern coast of Tanzania along the Indian Ocean. It is about 45 kilometers (28 miles) from Dar es Salaam, the country's biggest city and commercial center of Tanzania. It is predominantly rural with spots of sub-urban centres along the highway to the Southern regions and to Mozambique. The district-estimated population is about 223,000 with 46.6% in the age range between 18 and 65 years^29^. Administratively, the district has 25 homogeneous wards in terms of the climate and economic activities. There are two main seasons, the wet-humid and dry-hot with average day temperature ranging between 20°C/68°F and 32°C/89.6°F in the months of July/August and January/December respectively.

### Study population

The study sample included de facto resident adults aged between 18 and 65 years in the study area. In addition to the eligibility criteria, we included only individuals able to speak English or Kiswahili, a common language to almost everybody in Tanzania.

### Sample size estimation

We used a formula, n = Z2p(1-p)/E2, by Kish^31^ to estimate the sample for the study. Z being the critical value of the normal distribution at 95% confidence level, p is an estimate proportion of the population with good knowledge about DF. Although the available estimate of knowledge on dengue was among healthcare workers in NorthEastern Tanzania^32^ was about 75%, we estimated this proportion to be about 25% in the general population of Mkuranga. The margin of error, E, was set at 5%. We also adjusted for possible sampling errors villages by the design effect of 1.5 and for a 5% possibility of non-participation. These parameters yielded a minimum sample of 455.

### Sampling procedure

We selected Pwani region purposefully and randomly selected Mkuranga district. From the 18 wards of Mkuranga district, we randomly selected at least 20% of the wards that will form a representative sample of the other wards. Selected wards were: Dondo, Kitomondo, Mbezi and Mipeko. The total population in the four wards is about 21771 (9.8% of the District's population)^29^. We randomly selected one village from each ward. We considered all households in the village to be eligible. Inclusion criteria were an adult (aged at least 18 years), able to communicate in Kiswahili language and not too ill to participate. From a list of eligible adults in a household, we randomly selected and interviewed one adult.

### Study tools

The study team included a supervisor and an interviewer who was an experienced person with medical background with some skills in interviewing techniques. We used an interview form organized in three sections: background information of the respondent, knowledge and attitude sections. We assessed knowledge in terms of symptoms, transmission and preventive methods. There were seven items to assess their attitudes with focus on prevention, severity of illness and health seeking behaviors. We first developed the tool in English, translated into the national language, Kiswahili, but later re-translated into English to make sure we maintained the original meaning. To assess the data collection procedure and the quality of the questions, we pre-tested the tool in the households in the nearby ward that was not earmarked for the study.

### Data processing and analysis

We used frequencies to summarize categorical variables. In order to have the index of knowledge, we scored each correct item with 1 point. Therefore, we scored a question on symptoms knowledge with 14 points, 8 points on transmission; 10 points on prevention and on health seeking behavior. We scaled an individual who scored between 0 and 11 points having a low knowledge, 12 to 23 points having medium knowledge and between 24 to 34 points as having high knowledge. We used a five-point Likert scale ranging between (1 =strongly agree; 5 =strongly disagree) to assess attitudes towards DF based on six items. During data processing, a respondent could potentially score from six to 30 points. Since low scores signified a negative attitude and high scores a positive attitude, a cut-off point was to all individuals scoring above 80% of the scores labeled as having positive attitude^33^. Although we did not use any standard tool when measuring knowledge and attitude of study participants about DF, we benchmarked with other studies and considering the study population, we were confident of the inter-rater reliability and its internal consistency. The internal consistency assessment using Cronbach's alpha was moderate reliable (α=0.581)34. We used Statistical Package for Social Sciences (SPSS), version 20, in all data analyses.

## Results

### Description of study participants

We recruited 441 adults aged between 18 and 65 (participation rate = 96.7%). A shortfall of 14 (3.1%) participants was due to unwillingness to participate mainly because of the selected potential participant claiming too busy with harvesting the crops. Their mean age of the study participants was 33.7 (SD=12.0) years. The majority, 251 (56.9%) were females, 192 (43.5), younger than 30 years; 280 (63.5%) were married and 269 (61.0%) had some primary education (Table 1).

**Table 1:** Socio-demographic characteristics of the study participants (n = 441)

Characteristics	Number (%)
Sex	
Male	190
Female	(43.1)
Age group (years)	251
< 20	(56.9)
20 – 24	
25 – 29	32 (7.3)
30 – 34	103
35 – 39	(23.4)
40 – 44	57 (12.9)
45 – 49	53 (12.0)
50+	60 (13.6)
Marital status	49 (11.1)
Single	34 (7.7)
Married	53 (12.0)
Cohabiting	
Divorced	116
Separated	(26.3)
Widow	280
Education status	(63.5)
Never in school	8 (1.8)
Primary*	14 (3.2)
Secondary*	9 (2.0)
Above secondary	14 (3.2)
Source of income	
Agriculture	55 (12.5)
Petty business	269
Salaried	(61.0)
Unemployed	93 (21.1) 24 (5.4)
	174 (39.5) 126 (28.5) 28 (6.3) 70 (15.9)

* Complete or incomplete

### Knowledge about dengue fever

**Figure 2:**
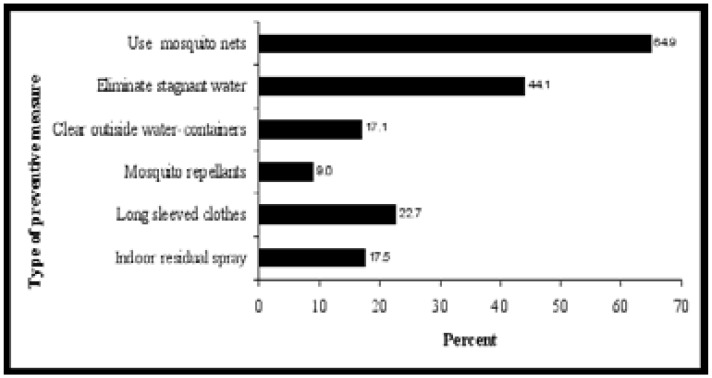
Preventive measures against mosquitoes that transmit dengue fever

Among all respondents, 431 (97.7%) reported ever heard of DF. In table 2, we present the average scores for each domain of knowledge assessment. All study participants thought DF is an airborne or food-borne disease and 97 (22.6%) thought one can get the virus through blood transfusion. Almost one in four of the participants thought the mosquito that transmits DF virus attacks at night (Figure 2).

**Table 2:** Average score of study participants on knowledge of symptoms, transmission, prevention and health seeking about dengue fever (n=431)

Knowledge domain	Total score	Average score	Percentage of knowledge
Symptoms	14	2.0 (SD = 1.4)	14.3
Transmission	8	2.6 (SD = 0.6)	32.5
Prevention	10	2.8 (SD = 1.7)	28.0
Health seeking	2	1.6 (SD = 0.6)	80.0
**Total**	34	9.5 (SD = 3.1)	2.4

Overall, the majority, 220 (77.7%) had a low knowledge and 63 (22.3%) had medium knowledge on DF. None of the study participants had a high knowledge. Among all participants, 39 (11.5%) thought dengue is an airborne disease and 347 (80.5%) considered the disease to be curable. Mean knowledge about dengue transmission was 2.6 (SD=0.6) that was statistically significant higher (p < 0.01) than the mean score knowledge about symptoms, 2.0 (SD=1.4).

### Attitude towards dengue fever

Only 76 (19.2%) had positive attitude towards DF prevention, severity of the illness and health seeking behaviour. Very few, 29 (6.7%) study participants considered DF a very serious illness, 177 (41.0%) said DF is a non-curable disease therefore no need of going to the health facility and 176 (44.6%) said the only possibility to control DF was when the government kills all mosquitoes in the community.

### Practices against contracting dengue virus

**Figure 3:**
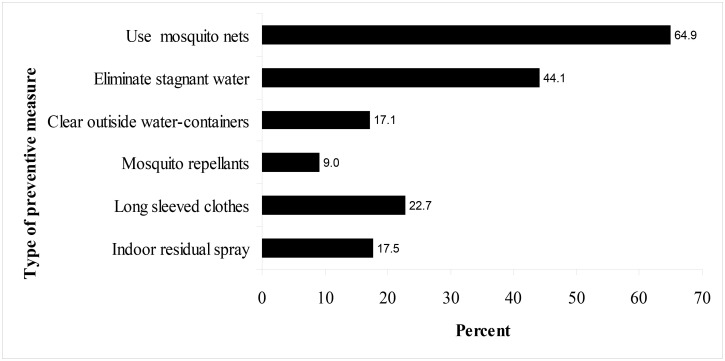
Preventive measures against mosquitoes that transmit dengue fever

Among all study participants, 209 (47.4%) reported they practice several measures to prevent themselves against dengue virus. Figure 3 shows different preventive measures against DF. The majority, 137 (64.9%) mentioned netting and 93 (44.1%) eliminating stagnant waters around their houses as measures against DF.

## Discussion

This study aimed to assess knowledge, attitude and practices among adult members of the rural communities in one region of Tanzania. Like for many other diseases, these three components have a major contribution when planning to control the spread of DF in the community^35^. In this study, knowledge about dengue fever is poor and the majority have negative attitude towards dengue fever in terms of preventive methods, severity of the disease and in health seeking behavior. Most of the cited practices, for example more than 60% indicating use of sleeping mosquito nets, are not against mosquito bites responsible for dengue virus, therefore not specifically targeting *Aedes aegypti*.

Correct knowledge of dengue transmission, symptoms and good practices are essential in controlling DF before and when there is an outbreak^36^. More than three quarters of the adult communities have low knowledge about dengue transmission, symptoms and preventive mechanisms. However, lower knowledge on symptoms than about the disease transmission has been recently reported in Malaysia. Similarly, the knowledge score of DF in Vietnam was 24% (4.6/19) that was lower than the score in Tanzania, 28% (9.5/34)^37^. Source of low knowledge, especially about transmission, maybe attributed to failure to distinguish between malaria and dengue fevers^38^. Since both dengue and malaria have similar etiologies, community members especially those with low or no education are more likely to confuse some transmission mechanism and prevention. This finding was not surprising because even in the Caribbean, Malaysia and Vietnam, education level was associated with higher knowledge of dengue^37^,^39^,^40^. Furthermore, community members in a neighbouring region of Morogoro perceive most fevers are due to malaria^41^. This finding constitutes one of the evidences for poor knowledge about dengue transmission mechanism among community members.

Vector control measures mentioned in this study that includes netting, indoor residual spray and use of mosquito repellant are also similar preventive measures against *Anopheles* mosquitoes responsible for malaria. Therefore, although correct, but respondents could have be referring to control measures against malria than DF.

In this study, only about 20% of the respondents have positive attitude towards DF prevention. In Ethiopia, slightly higher than 20% of health care professionals had positive attitude^42^. Therefore, a smaller proportion of adults having positive attitude is an indication of a poor understanding of DF. Good knowledge about DF may not necessarily imply positive attitude on the disease^43^.

This study has several potential limitations. Although we used well-trained research assistants with medical background, it is possible that their outlook (language and physical presentation) influenced respondents offering socially desirable responses leading to desirability bias especially when giving self-report on attitude about DF. We could have reduced this kind of bias by using a self-administered questionnaire, but we were not able to make a prior confirmation of the community literacy level necessary for such data collection technique. We conducted this study in rural settings in Pwani Region. Although technically inference from this study only applies to the study area, knowledge, attitude and practices towards DF may not significantly differ from other rural populations of Tanzania. However, in-depth qualitative data may give more insight on the subject matter. Furthermore, we propose a study to assess people's views on the development of vaccine towards prevention of dengue in the future.

## Conclusion

Rural adults in Pwani Region of Tanzania with low education status have low knowledge about DF transmission, symptoms and practices. Lack of proper knowledge about disease transmission, symptoms and preventive measures put the population at high risk of contracting the virus. There is need to create or improve friendly, correct and simple information, education and educational messages for the rural populations. These messages could be channeled to the community through the existing platforms. Examples of avenues to convey these messages include available trained community educators, social media, schools and health care providers. Finally, we propose the increased efforts in the development and eventually availability of vaccine against DF towards prevention and elimination of the disease in humans.

## List of abbreviations

DF: Dengue fever
SD: Standard deviation

## References

[1] Elsinga J, van der Veen HT, Gerstenbluth I, Burgerhof JGM, Dijkstra A, Grobusch MP (2017). Community participation in mosquito breeding site control: an interdisciplinary mixed methods study in Curaçao. Parasit Vectors.

[2] World Health Organization (WHO) (2012). Dengue and Severe Dengue – Fact sheet N°117.

[3] Zhang H, Zhou YP, Peng HJ, Zhang XH, Zhou FY, Liu ZH, Chen XG (2014). Predictive symptoms and signs of severe dengue disease for patients with dengue fever: a meta-analysis. Biomed Res Int.

[4] Kajeguka DC, Kaaya RD, Mwakalinga S, Ndossi R, Ndaro A, Chilongola JO (2016). Prevalence of dengue and chikungunya virus infections in north-eastern Tanzania: a cross sectional study among participants presenting with malaria-like symptoms. BMC Infect Dis.

[5] Peter JV, Sudarsan TI, Prakash JA, Varghese GM (2015). Severe scrub typhus infection: Clinical features, diagnostic challenges and management. World J Crit Care Med.

[6] Halstead SB (2008). Dengue virus-mosquito interactions. Annu Rev Entomol.

[7] Tran BX, Thu Vu G, Hoang Nguyen L, Tuan Le Nguyen A, Thanh Tran T, Thanh Nguyen B, Phuong Thi Thai T, Latkin CA, Ho CSH, Ho RCM (2018). Cost-of-Illness and the Health-Related Quality of Life of Patients in the Dengue Fever Outbreak in Hanoi in 2017. Int J Environ Res Public Health.

[8] Chaloemwong J, Tantiworawit A, Rattanathammethee T, Hantrakool S, Chai-Adisaksopha C, Rattarittamrong E, Norasetthada L (2018). Useful clinical features and hematological parameters for the diagnosis of dengue infection in patients with acute febrile illness: a retrospective study. BMC Hematol.

[9] Nealon J, Taurel AF, Capeding MR (2016). Symptomatic Dengue Disease in Five Southeast Asian Countries: Epidemiological Evidence from a Dengue Vaccine Trial. PLoS Negl Trop Dis.

[10] Furuya-Kanamori L, Liang S, Milinovich G, Soares Magalhaes RJ, Clements AC, Hu W, Brasil P, Frentiu FD, Dunning R, Yakob L (2016). Co-distribution and co-infection of chikungunya and dengue viruses. BMC Infect Dis.

[11] Getachew D, Tekie H, Gebre-Michael T, Balkew M, Mesfin A (2015). Breeding Sites of Aedes aegypti: Potential Dengue Vectors in Dire Dawa, East Ethiopia. Interdiscip Perspect Infect Dis.

[12] Mboera LE, Mweya CN, Rumisha SF, Tungu PK, Stanley G, Makange MR (2016). The Risk of Dengue Virus Transmission in Dar es Salaam, Tanzania during an Epidemic Period of 2014. PLoS Negl Trop Dis.

[13] Were F (2012). The dengue situation in Africa. Paediatr Int Child Health.

[14] Weetman D, Kamgang B, Badolo A, Moyes CL, Shearer FM, Coulibaly M (2018). Aedes Mosquitoes and Aedes-Borne Arboviruses in Africa: Current and Future Threats. Int J Environ Res Public Health.

[15] Leta S, Beyene TJ, De Clercq EM, Amenu K, Kraemer MUG, Revie CW (2018). Global risk mapping for major diseases transmitted by Aedes aegypti and Aedes albopictus. Int J Infect Dis.

[16] Ferede G, Tiruneh M, Abate E, Wondimeneh Y, Damtie D, Gadisa E (2018). A serologic study of dengue in northwest Ethiopia: suggesting preventive and control measures. PLoS Negl Trop Dis.

[17] Eldigail MH, Adam GK, Babiker RA, Khalid F, Adam IA, Omer OH (2018). Prevalence of dengue fever virus antibodies and associated risk factors among residents of El-Gadarif state, Sudan. BMC Public Health.

[18] Ridde V, Agier I, Bonnet E, Carabali M, Dabiré KR, Fournet F (2016). Presence of three dengue serotypes in Ouagadougou (Burkina Faso): research and public health implications. Infect Dis Poverty.

[19] Simo FBN, Bigna JJ, Kenmoe S, Ndangang MS, Temfack E, Moundipa PF, Demanou M (2019). Dengue virus infection in people residing in Africa: a systematic review and meta-analysis of prevalence studies. Sci Rep.

[20] Vairo F, Nicastri E, Meschi S, Schepisi MS, Paglia MG, Bevilacqua N, Mangi S (2012). Seroprevalence of dengue infection: a cross-sectional survey in mainland Tanzania and on Pemba Island, Zanzibar. Int J Infect Dis.

[21] Mboera LE, Mweya CN, Rumisha SF, Tungu PK, Stanley G, Makange MR, Misinzo G, De Nardo P, Vairo F, Oriyo NM (2016). The Risk of Dengue Virus Transmission in Dar es Salaam, Tanzania during an Epidemic Period of 2014. PLoS Negl Trop Dis.

[22] Ward T, Samuel M, Maoz D, Runge-Ranzinger S, Boyce R, Toledo J, Velayudhan R, Horstick O (2017). Dengue data and surveillance in Tanzania: a systematic literature review. Trop Med Int Health.

[23] Okada K, Morita R, Egawa K, Hirai Y, Kaida A, Shirano M, Kubo H, Goto T, Yamamoto SP (2019). Dengue Virus Type 1 Infection in Traveler Returning from Tanzania to Japan, 2019. Emerg Infect Dis.

[24] Boillat-Blanco N, Klaassen B, Mbarack Z, Samaka J, Mlaganile T, Masimba J, Franco Narvaez L (2018). Dengue fever in Dar es Salaam, Tanzania: clinical features and outcome in populations of black and non-black racial category. BMC Infect Dis.

[25] Nguyen LH, Tran BX, Do CD, Hoang CL, Nguyen TP, Dang TT, Thu Vu G, Tran TT, Latkin CA, Ho CS, Ho RC (2018). Feasibility and willingness to pay for dengue vaccine in the threat of dengue fever outbreaks in Vietnam. Patient Prefer Adherence.

[26] WHO (2003). Guidelines for dengue surveilllance and mosquito control.

[27] Teixeira MG, Barreto ML (2009). Diagnosis and management of dengue. BMJ.

[28] Mohammed YA, Abdurashid IN (2019). Knowledge, attitude and practice towards dengue fever prevention and associated factors among public health sector healthcare professionals: in Dire Dawa, eastern Ethiopia. Risk Manag Healthc Policy.

[29] National Bureau of Statistics (NBS) [Tanzania] and ORC Macro (2014). Tanzania Population and Housing Census 2012.

[30] Torell, Elin and Aviti Mmochi (2006). Mkuranga Governance Baseline.

[31] Kish L (1965). Survey sampling.

[32] Saringe S, Kajeguka DC, Kagirwa DD, Mgabo MR, Emidi B (2019). Healthcare workers knowledge and diagnostic practices: a need for dengue and chikungunya training in Moshi Municipality, Kilimanjaro Tanzania. BMC Res Notes.

[33] Dhimal M, Aryal KK, Dhimal ML, Gautam I, Singh SP, Bhusal CL, Kuch U (2014). Knowledge, attitude and practice regarding dengue fever among the healthy population of highland and lowland communities in central Nepal [published correction appears in PLoS One. 2014;9(10):e110605]. PLoS One.

[34] Tavakol M, Dennick R (2011). Making sense of Cronbach's alpha. Int J Med Educ.

[35] Alyousefi TAA, Abdul-Ghani R, Mahdy MAK, Al-Eryani SMA, Al-Mekhlafi AA, Raja YA (2016). A household-based survey of knowledge, attitudes and practices towards dengue fever among local urban communities in Taiz Governorate, Yemen. BMC Infect Dis.

[36] Yboa BC, Labrague LJ (2013). Dengue knowledge and preventive practices among rural residents in Samar Province, Philippines. Am J Public Health Res.

[37] Nguyen HV, Than PQT, Nguyen TH, Vu GT, Hoang CL, Tran TT, Truong NT (2019). Knowledge, Attitude and Practice about Dengue Fever among Patients Experiencing the 2017 Outbreak in Vietnam. Int J Environ Res Public Health.

[38] Epelboin L, Boullé C, Ouar-Epelboin S, Hanf M, Dussart P, Djossou F (2013). Discriminating malaria from dengue fever in endemic areas: clinical and biological criteria, prognostic score and utility of the C-reactive protein: a retrospective matched-pair study in French Guiana. PLoS Negl Trop Dis.

[39] Diaz-Quijano FA, Martínez-Vega RA, Rodriguez-Morales AJ, Rojas-Calero RA, Luna-González ML, Díaz-Quijano RG (2018). Association between the level of education and knowledge, attitudes and practices regarding dengue in the Caribbean region of Colombia. BMC Public Health.

[40] Lugova H, Wallis S J (2017). Cross-Sectional Survey on the Dengue Knowledge, Attitudes and Preventive Practices Among Students and Staff of a Public University in Malaysia. Community Health.

[41] Chipwaza B, Mugasa JP, Mayumana I, Amuri M, Makungu C, Gwakisa PS (2014). Community knowledge and attitudes and health workers' practices regarding non-malaria febrile illnesses in eastern Tanzania. PLoS Negl Trop Dis.

[42] Yusuf AM, Ibrahim NA (2019). Knowledge, attitude and practice towards dengue fever prevention and associated factors among public health sector health-care professionals: in Dire Dawa, eastern Ethiopia. Risk Manag Healthc Policy.

[43] Mohammed AA, Mohammed FB, Anis RA, Mohammed AA, Lavannya RP, Bavani K, Kavitha B (2017). Knowledge, attitude and practice towards dengue fever among patients in Hospital Taiping. MJPHM.

